# *ASXL1* mutation correction by CRISPR/Cas9 restores gene function in leukemia cells and increases survival in mouse xenografts

**DOI:** 10.18632/oncotarget.6392

**Published:** 2015-11-26

**Authors:** Simona Valletta, Hamid Dolatshad, Matthias Bartenstein, Bon Ham Yip, Erica Bello, Shanisha Gordon, Yiting Yu, Jacqueline Shaw, Swagata Roy, Laura Scifo, Anna Schuh, Andrea Pellagatti, Tudor A. Fulga, Amit Verma, Jacqueline Boultwood

**Affiliations:** ^1^ Bloodwise Molecular Haematology Unit, Nuffield Division of Clinical Laboratory Sciences, Radcliffe Department of Medicine, University of Oxford and BRC Blood Theme, NIHR Oxford Biomedical Centre, Oxford University Hospital, Oxford, UK; ^2^ Albert Einstein College of Medicine, Bronx, NY, USA; ^3^ NIHR Biomedical Research Centre, University of Oxford, Oxford, UK; ^4^ Weatherall Institute of Molecular Medicine, Radcliffe Department of Medicine, University of Oxford, John Radcliffe Hospital, Oxford, UK

**Keywords:** ASXL1, CRISPR, chronic myeloid leukemia, mutation correction, tumor suppressor

## Abstract

Recurrent somatic mutations of the epigenetic modifier and tumor suppressor *ASXL1* are common in myeloid malignancies, including chronic myeloid leukemia (CML), and are associated with poor clinical outcome. CRISPR/Cas9 has recently emerged as a powerful and versatile genome editing tool for genome engineering in various species. We have used the CRISPR/Cas9 system to correct the *ASXL1* homozygous nonsense mutation present in the CML cell line KBM5, which lacks ASXL1 protein expression. CRISPR/Cas9-mediated *ASXL1* homozygous correction resulted in protein re-expression with restored normal function, including down-regulation of Polycomb repressive complex 2 target genes. Significantly reduced cell growth and increased myeloid differentiation were observed in *ASXL1* mutation-corrected cells, providing new insights into the role of ASXL1 in human myeloid cell differentiation. Mice xenografted with mutation-corrected KBM5 cells showed significantly longer survival than uncorrected xenografts. These results show that the sole correction of a driver mutation in leukemia cells increases survival *in vivo* in mice. This study provides proof-of-concept for driver gene mutation correction via CRISPR/Cas9 technology in human leukemia cells and presents a strategy to illuminate the impact of oncogenic mutations on cellular function and survival.

## INTRODUCTION

The clustered regularly interspaced short palindromic repeats (CRISPR)–CRISPR-associated protein 9 (Cas9) (CRISPR/Cas9) is a microbial adaptive immune system that uses RNA-guided nucleases to cleave foreign genetic elements. This system has recently emerged as a powerful and versatile tool for genome engineering in various species, and can be used to correct gene mutations in cells via genome editing [1–4]. The system employs the type-II prokaryotic CRISPR adaptive immune system, which uses a guide RNA to target the Cas9 nuclease to a specific 20 nt genomic sequence upstream of a “protospacer adjacent motif” (PAM), which can take the form of NGG or NAG [5]. Cas9 induces double-stranded DNA breaks which are repaired either by imperfect non-homologous end joining (NHEJ) to generate indels [6] or, if a repair template is provided, by homology directed repair (HDR) [3].

The CRISPR/Cas9 system has been used to perform targeted genome engineering in human cells [7, 8], including genetic correction [9] and introduction of large chromosomal deletions or inversions [4, 7]. It has been recently demonstrated that the CRISPR/Cas9 system can be used for rapid genome editing in mouse embryos and human stem cells in culture [3, 10–12]. For example, this strategy has been employed to correct the CFTR locus in cultured intestinal stem cells of patients with Cystic Fibrosis [9]. This study demonstrated the feasibility of gene correction in primary adult stem cells derived from patients with a monogenic hereditary defect, thus paving the way for future gene therapy approaches [9]. In another study, CRISPR-Cas9–mediated correction of a *Fah* mutation was performed in hepatocytes in a mouse model of the human disease hereditary tyrosinemia [12]. Expansion of Fah-positive hepatocytes rescued the body weight loss phenotype [12].

Given its successful application for gene correction in cultured cells from patients with monogenic hereditary defects, we reasoned that the CRISPR/Cas9 system could be employed to correct acquired gene mutations found in human leukemia cells.

Additional sex combs-like 1 (ASXL1), a polycomb family member, plays an important role in epigenetic regulation, activating or repressing the transcription of genes involved in either differentiation or proliferation through its effect on histone methylation marks. ASXL1 is involved in the recruitment of the Polycomb repressive complex 2 (PRC2) to specific loci [13, 14]. *ASXL1* is frequently mutated in a range of myeloid malignancies, including the myelodysplastic syndromes (MDS), chronic myelomonocytic leukemia (CMML), and acute myeloid leukemia [15, 16]. We were the first to report that mutations of *ASXL1* occur in chronic myeloid leukemia (CML) [17], and *ASXL1* mutations have been associated with disease progression and blast crisis in CML [18, 19]. *ASXL1* mutations are strongly associated with a poor prognosis in these myeloid disorders [20]. *ASXL1* mutations are typically found in exon 12, within a hotspot of mutations (including frameshift and nonsense mutations), and are considered to be loss-of-function mutations [21, 22]. A recent report has demonstrated that nonsense and frameshift mutations result in loss of ASXL1 expression, consistent with ASXL1 functioning as a tumor suppressor [13]. The mechanisms by which *ASXL1* mutations contribute to myeloid transformation are becoming increasingly clear [13] but are not yet fully understood.

In this study we have used CRISPR/Cas9-mediated HDR to correct the homozygous *ASXL1* mutation found in the CML KBM5 cell line [13] and we have performed functional studies to determine whether the wild-type function of ASXL1 was restored following gene correction. We then performed *in vivo* experiments to determine the impact of *ASXL1* mutation correction on survival in mouse xenografts.

## RESULTS

### Correction of *ASXL1* mutation in KBM5 cells using CRISPR/Cas9 system

**Figure 1:**
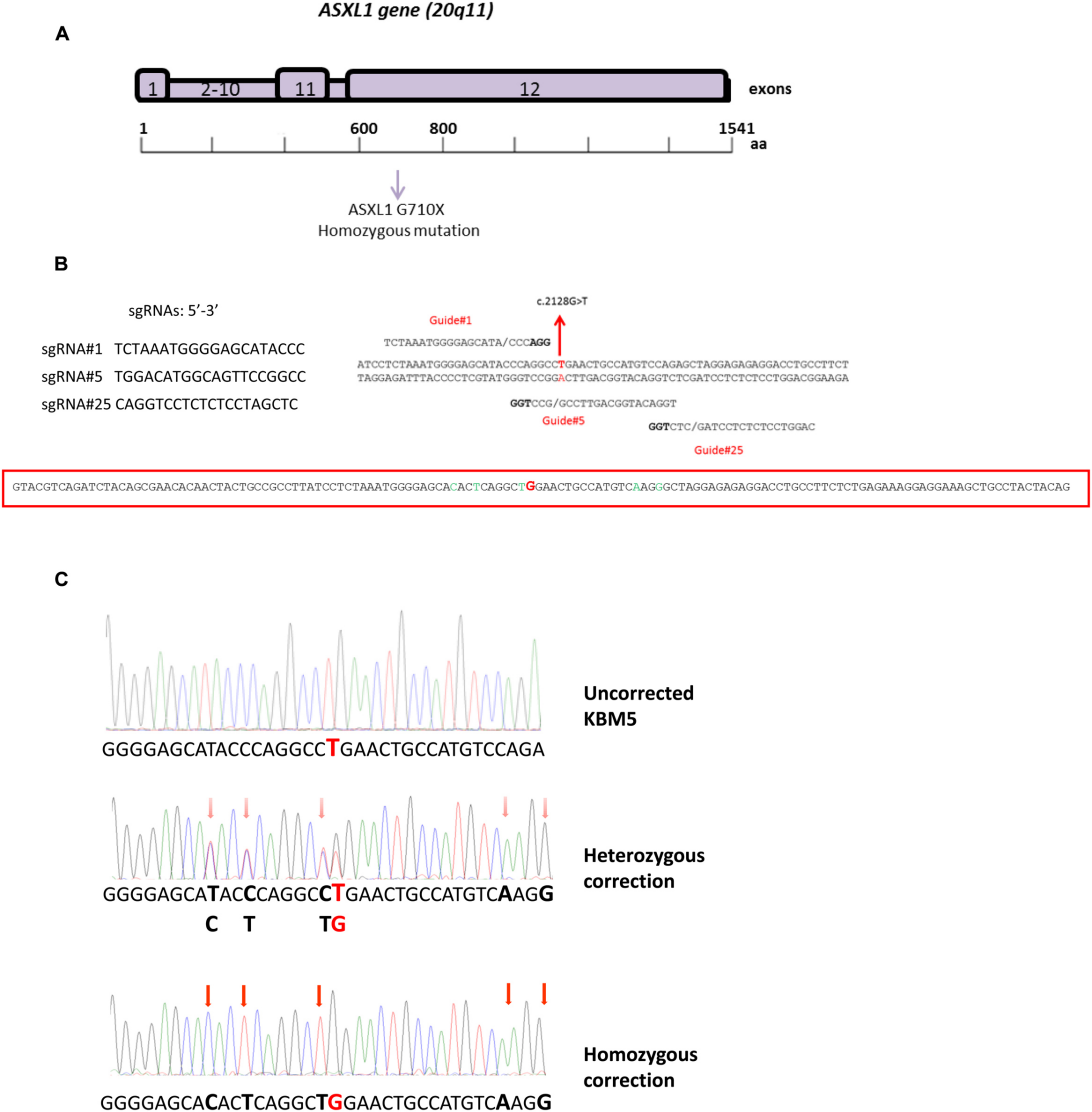
CRISPR/Cas9-mediated correction of *ASXL1* mutations in the CML cell line KBM5 (**A**) Structure of the *ASXL1* gene. *ASXL1* maps to the chromosome region 20q11 and comprises 12 exons. The *ASXL1* G710X mutation found in KBM5 cells is located in exon 12. (**B**) Design of sgRNAs and ssODN repair template used for the CRISPR/Cas9-mediated *ASXL1* mutation correction. Left-hand side: sequences of three sgRNAs (sgRNA#1, sgRNA#5, sgRNA#25), identified using the crispr.mit.edu online resource. Right-hand side: alignment of the three sgRNAs to the genomic region containing the *ASXL1* mutation (indicated in red) in KBM5 cells. Each site comprises 20 nt followed by a trinucleotide (5′-NGG-3′) protospacer adjacent motif (PAM), highlighted in bold, which is required for Cas9 activity (DNA double-strand break). Bottom: sequence of the ssODN used as repair template in the HDR. The G nucleotide, which corrects the mutated T nucleotide in KBM5 cell line, is highlighted in red; five silent nucleotides changes (i.e. not causing amino acid changes in the resulting ASXL1 protein) were introduced in the ssODN sequence (highlighted in green) to avoid undesired Cas9 activity in mutation-corrected cells. (**C**) Evaluation of *ASXL1* mutation correction in KBM5 cells using Sanger sequencing. Top trace: sequencing trace showing the presence of homozygous *ASXL1* point mutation (GGA > TGA, p.G710X); the mutated nucleotide (G > T) is highlighted in red. Middle trace: representative sequencing trace showing heterozygous correction of the *ASXL1* mutation; KBM5 clones with heterozygous correction retain the mutant allele (G / T), highlighted in red in the sequencing trace. Bottom trace: representative sequencing trace showing homozygous correction (G) of the *ASXL1* mutation. The red arrows in the middle and bottom panel indicate the silent nucleotides changes that were introduced in the ssODN sequence.

The human myeloid leukemia cell line KBM5 (derived from a CML patient in blast phase) was chosen for this study as it lacks wild-type ASXL1 protein expression, due to a homozygous point mutation (c.2128G > T, p.G710X) in the *ASXL1* gene that creates a premature termination codon [13] (Figure 1A). We confirmed the presence of the homozygous *ASXL1* G710X mutation (variant allele frequency 99.9) in KBM5 cells using a targeted next-generation sequencing myeloid gene panel [23] which also identified a homozygous *TP53* mutation (R273H, variant allele frequency 99.4).

We used three custom-designed synthetic single guide RNAs (sgRNAs) targeting the genomic region overlapping the *ASXL1* mutation observed in KBM5 cells (Figure 1B). Each sgRNA was cloned into the pX458 [pSpCas9(BB)-2A-GFP] vector which also encodes the Cas9 nuclease [3]. A 140-nt single-stranded DNA oligonucleotide (ssODN) containing the wild-type G nucleotide was used as template for HDR (Figure 1B). The vector and ssODN were transfected into KBM5 cells, and single cells expressing GFP were sorted by FACS into individual wells in 96-well plates and expanded in culture. Successful HDR-mediated mutation correction was assessed by Sanger sequencing.

We sequenced 1,027 colonies and each of the three sgRNAs yielded 2%, 0.46% and 1.4% heterozygous precise correction. Importantly, we observed homozygous precise correction (i.e. all *ASXL1* mutated alleles present) for two of the three sgRNAs with a yield of 1.63% and 1.13% (Figure 1C). The frequency of correction that we obtained is consistent with previous studies using the CRISPR/Cas9 system (0.1–3.3%) for precise genome editing through HDR [4, 24].

Since the CRISPR/Cas9 system can potentially generate undesired off-target effects [9], we analyzed the top five predicted off-targets sites for each of the three sgRNAs as well as the entire coding sequence of the *ASXL1* gene using Sanger sequencing [10]. No sequence alterations were detected in any of the off-target sites examined or in the *ASXL1* gene in all *ASXL1* mutation-corrected clones identified, suggesting high specificity of CRISPR/Cas9 system in our experiments.

### Restoration of ASXL1 protein expression

**Figure 2:**
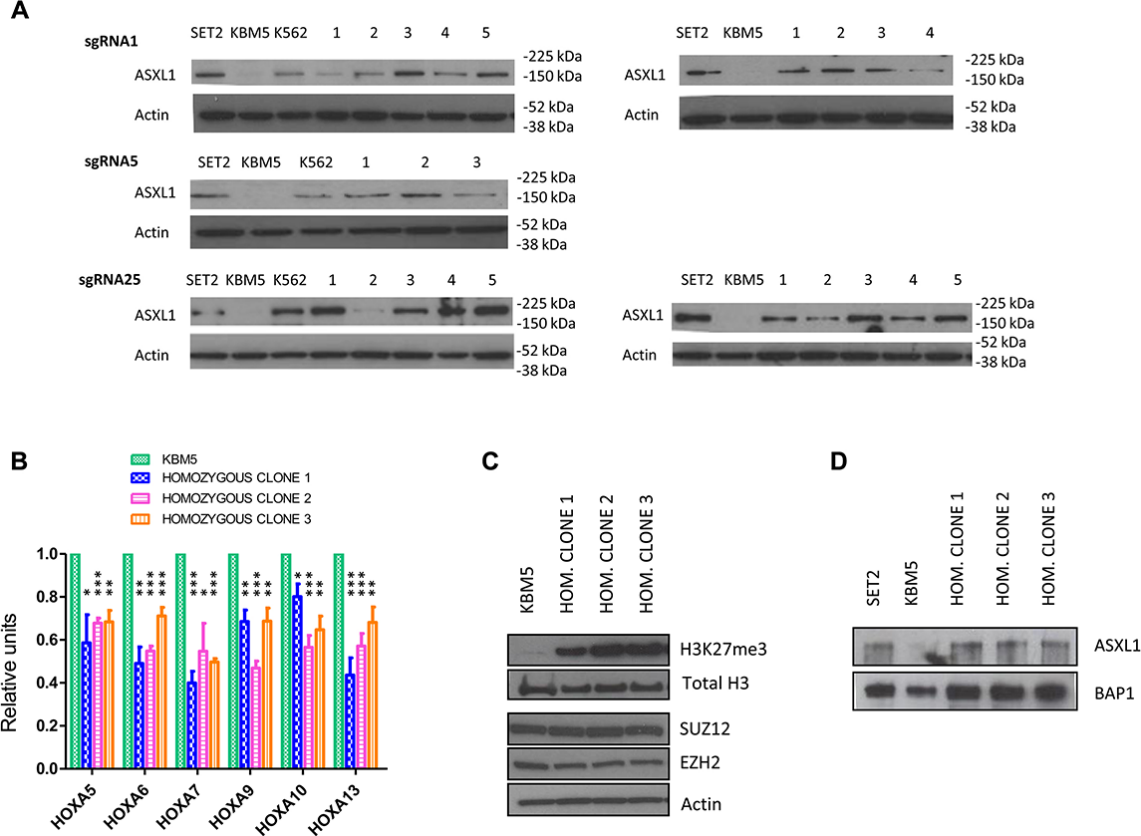
Functional effects of CRISPR/Cas9-mediated *ASXL1* mutation correction (**A**) Evaluation of ASXL1 protein expression by Western blotting. Left-hand side: ASXL1 protein expression in the SET2 leukemia cell line (wild-type for *ASXL1*), uncorrected KBM5 cells, the K562 leukemia cell line (carrying the Y591X heterozygous *ASXL1* mutation), and KBM5 clones (labeled 1–5) with heterozygous precise correction of the *ASXL1* mutation. Right-hand side: ASXL1 protein expression in the SET2 leukemia cell line (wild-type for *ASXL1*), uncorrected KBM5 cells, and KBM5 clones (labeled 1–5) with homozygous precise correction of the *ASXL1* mutation. β-actin was used as loading control. (**B**) Evaluation of the expression levels of *HOXA* genes using quantitative real-time PCR (q-RT-PCR). The expression levels of *HOXA5, 6, 7, 9, 10* and *13* were measured in three *ASXL1* homozygous corrected KBM5 clones compared with uncorrected cells. The results shown were obtained from six independent experiments for each clone. The values in *ASXL1* homozygous corrected cells are relative to the uncorrected cells. Bar graphs show mean + standard error of the mean (s.e.m.) (* = *P* < 0.05, ** = *P* < 0.01, *** = *P* < 0.001, paired *t*-test). (**C**) Evaluation of H3K27me3 levels and expression of PRC2 components by Western blotting in uncorrected KBM5 cells and three KBM5 clones with homozygous correction of the *ASXL1* mutation. H3K27me3 levels and total H3 levels were evaluated using purified histone fractions. The expression levels of two PRC2 components (EZH2, SUZ12) were determined using whole cell lysates. β-actin was used as loading control. (**D**) Immunoprecipitation of BAP1 in the SET2 leukemia cell line (wild-type for ASXL1), uncorrected KBM5 cells, and three KBM5 clones with homozygous correction of the *ASXL1* mutation. The BAP1 protein fraction was immunoprecipitated using a BAP1 antibody and stained for ASXL1 and BAP1.

Homozygous nonsense *ASXL1* mutations have been shown to result in loss of ASXL1 protein expression in myeloid leukemia cells [13]. We therefore investigated whether *ASXL1* mutation correction resulted in restoration of protein expression in KBM5 cells. We performed Western blot analysis and, as expected, uncorrected KBM5 cells had no detectable ASXL1 protein expression, compared to the myeloid cell line SET2 which is wild-type for ASXL1 (Figure 2A). Importantly, CRISPR/Cas9-based mutation correction resulted in full-length ASXL1 protein expression in both heterozygous and homozygous corrected KBM5 clones (Figure 2A). These results confirm that *ASXL1* mutation correction results in expression of the ASXL1 protein in leukemia cells.

### Effects on Polycomb repressive complex 2 function

The Polycomb repressive complex 2 (PRC2) is composed of three core subunits, SUZ12, EED, and either EZH1 or EZH2, that act through epigenetic modification of chromatin structure to maintain genes in a repressed state [25]. ASXL1 plays an important role in the recruitment of the PRC2 to specific target loci, including the HOXA cluster [26]. Loss of ASXL1 is associated with increased expression of *HOXA* genes, including *HOXA5-9*, contributing to myeloid transformation [13]. In order to assess whether *ASXL1* mutation correction resulted in decreased expression levels of *HOXA* genes, we performed quantitative real-time PCR of multiple *HOXA* members and observed significant down-regulation of *HOXA5, 6, 7, 9, 10* and *13* in *ASXL1* mutation-corrected KBM5 clones (Figure 2B).

Recent data have shown that ASXL1 interacts with the PRC2 components EZH2 and SUZ12, which play a critical role in the deposition of H3K27me3 (trimethylation of histone H3 lysine 27) histone repressive marks [26], and *ASXL1* mutations are associated with H3K27me3 loss in myeloid cells [13]. We evaluated H3K27me3 levels using western blots on purified histones from *ASXL1* mutation-corrected KBM5 clones. *ASXL1* mutation correction resulted in a marked increase in global H3K27me3 levels, whilst not affecting total H3 expression (Figure 2C) and preserving the expression of the core PRC2 members EZH2 and SUZ12 (Figure 2C). These data show that *ASXL1* mutation correction restores these repressive functions of the PRC2.

### Restoration of ASXL1-BAP1 interaction

Mammalian ASXL1 forms a protein complex *in vitro* with the tumour suppressor BAP1, a chromatin deubiquitinase that plays a role in the regulation of the cell cycle, cellular differentiation, and cell death [27]. This interaction is critical for the enzymatic activity of BAP1 and is reduced in cell lines carrying *ASXL1* mutations [13]. A BAP1-independent function of ASXL1 in regulating H3K27me3 through interactions with the PRC2 has been described [28]. BAP1 deficiency results in MDS/CMML-like disease *in vivo*, suggesting a central role for the ASXL1-BAP1 axis in myelopoiesis [29]. We therefore investigated whether the ASXL1-BAP1 association is restored by ASXL1 mutation correction. Immunoprecipitation studies showed that the interaction between ASXL1 and BAP1 was restored in *ASXL1* mutation-corrected KBM5 clones (Figure 2D).

### Cell growth and myeloid differentiation

**Figure 3:**
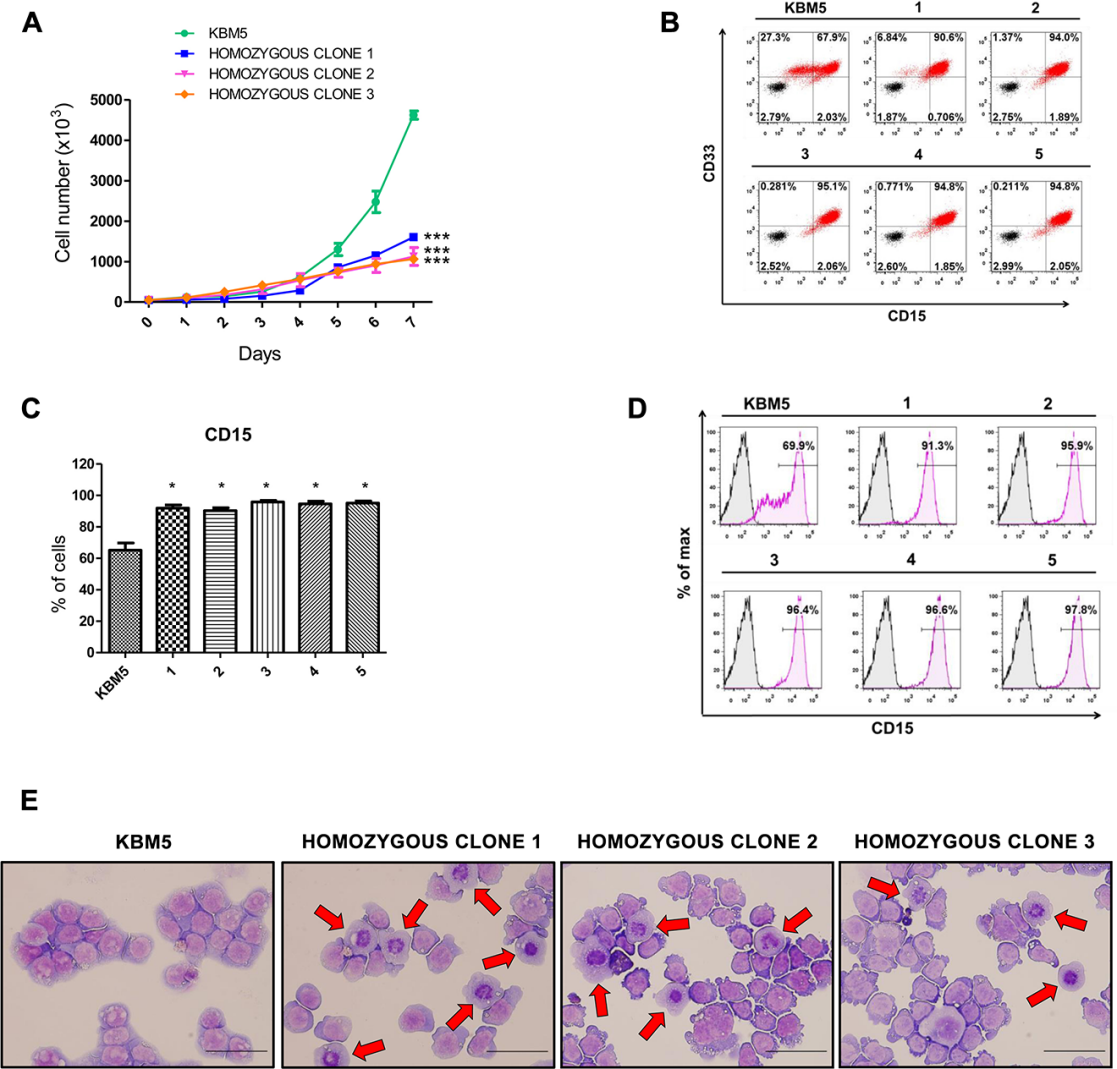
Effects of CRISPR/Cas9-mediated *ASXL1* mutation correction on cell growth and myeloid differentiation (**A**) Cell growth analysis. Viable cells were identified by trypan blue exclusion at different time points in culture. Uncorrected KBM5 cells and three KBM5 clones with homozygous correction of the *ASXL1* mutation were cultured at an initial density of 1 × 10^4^/mL and viable cells were counted over a 7-day period. The results shown were obtained from three independent experiments for each clone and represent mean ± s.e.m. (*** = *P* < 0.001, two-way ANOVA). (**B**) Flow cytometry contour plots showing the expression of the two myeloid surface markers CD33 and CD15 in KBM5 clones with homozygous correction of the *ASXL1* mutation (labeled 1–5) compared to uncorrected KBM5 cells. Cells stained with anti-CD33 and anti-CD15 antibodies are shown in red. A total of nine KBM5 clones with homozygous correction of the *ASXL1* mutation were analyzed (all showing a homogenous CD33^+^CD15^hi^ cell population) and data from five representative clones are shown. (**C**) Percentage of CD15^+^ cells in KBM5 clones with homozygous correction of the *ASXL1* mutation (labeled 1–5) compared to uncorrected KBM5 cells, assessed by flow cytometry. The results shown were obtained from six independent experiments for each clone. Bar graphs show mean ± s.e.m (* = *P* < 0.05, Wilcoxon signed-rank test). (**D**) Histogram plots showing expression of CD15^+^ cells in representative KBM5 clones with homozygous correction of the *ASXL1* mutation (labeled 1–5) compared to uncorrected KBM5 cells, assessed by flow cytometry. (**E**) Representative images of May-Grünwald/Giemsa stained uncorrected KBM5 cells and KBM5 clones with homozygous correction of the *ASXL1* mutation. The red arrows indicate cells with a decreased nucleus to cytoplasm ratio and condensed nuclei, which are signs of myeloid maturation. Scale bar indicates 25 μm.

It has been shown that enforced expression of wild-type ASXL1 in myeloid leukemia cell lines with homozygous nonsense *ASXL1* mutations resulted in growth suppression [13]. We investigated the effects of *ASXL1* mutation correction on cell growth, and we observed significant growth suppression in *ASXL1* mutation-corrected cells compared to uncorrected KBM5 cells (Figure 3A).

It is well known that the response of CML patients to the drug imatinib mesylate (a tyrosine kinase inhibitor that inhibits BCR-ABL) diminishes during disease progression [30]. In order to determine whether the response of KBM5 cells to imatinib was altered by the correction of the *ASXL1* mutation, we have cultured uncorrected and *ASXL1* mutation-corrected KBM5 cells with imatinib. Cells treated with imatinib showed a decrease in cell growth compared to the untreated counterpart, however no significant difference in cell growth was observed between KBM5 cells with heterozygous or homozygous correction of *ASXL1* and uncorrected cells treated with imatinib. These data are consistent with the notion that acquisition of *ASXL1* mutations per se may not drive tyrosine kinase inhibitor resistance in CML.

It has been shown that *ASXL1* mutations inhibit myeloid differentiation in murine cell lines *in vitro* and *in vivo* in mice [28]. The impact of *ASXL1* mutation on myeloid differentiation in human cells in culture has not been determined. The KBM5 cells used in this present study derive from a CML patient in myeloid blast crisis (> 20% immature blasts), and we have shown by flow cytometry that these cells express the surface markers CD33 (a pan-myeloid marker) and CD15 (a marker expressed on granulocytes and monocytes) (Figure 3B). CD15 showed a heterogeneous expression pattern in KBM5 cells, with the presence of CD33^+^CD15^−^, CD33^+^CD15^lo^ and CD33^+^CD15^hi^ cells (Figure 3B). Importantly, we observed a significant increase in the percentage of cells expressing CD15 (Figure 3C–3D), resulting in a homogenous CD33^+^CD15^hi^ cell population (Figure 3B), in *ASXL1* mutation-corrected KBM5 cells compared to uncorrected cells. Cytospin slides showed an increase in myeloid cell differentiation, with signs of early myeloid differentiation in mutation-corrected KBM5 cells compared to uncorrected cells (Figure 3E). These results show that *ASXL1* mutation correction promotes myeloid differentiation in KBM5 cells, providing new insights into the role of ASXL1 in human myeloid cell differentiation.

### Mouse xenografts

**Figure 4:**
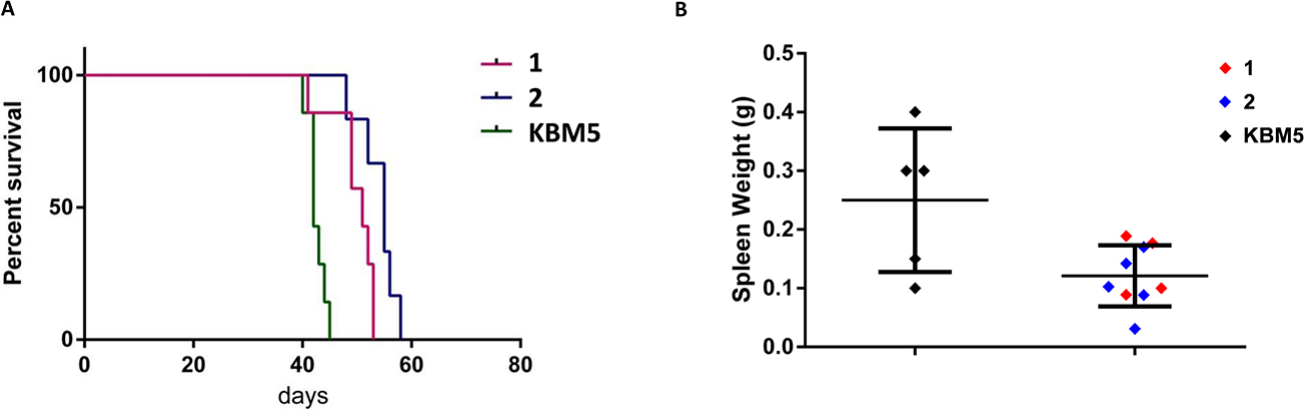
*In vivo* effects of CRISPR/Cas9-mediated *ASXL1* mutation correction in mouse xenografts (**A**) Kaplan-Meier plot showing the survival of NSG mice xenografted with uncorrected KBM5 cells (*n* = 7 mice) and two homozygous *ASXL1* mutation-corrected KBM5 clones (labeled 1–2) (clone 1, *n* = 7 mice; clone 2, *n* = 6 mice). (**B**) Spleen weights in NSG mice xenografted with uncorrected KBM5 cells and *ASXL1* mutation-corrected KBM5 clones (labeled 1–2).

To determine the effects of *ASXL1* correction *in vivo*, we xenografted uncorrected KBM5 cells and two homozygous *ASXL1* mutation-corrected KBM5 clones into non-obese diabetic–severe combined immunodeficiency–IL-2Rγ null (NSG) mice. Mice with corrected *ASXL1* demonstrated significantly longer survival than uncorrected control xenografts (*P* < 0.0001) (Figure 4A). Analysis of the spleens showed a significant decrease in spleen weight in mice xenografted with *ASXL1* mutation-corrected KBM5 clones (*P* = 0.016) (Figure 4B). These data show that correction of *ASXL1* mutation in leukemia cells increases survival *in vivo*.

## DISCUSSION

The CRISPR/Cas9 system has recently emerged as a powerful genome editing tool that has been used for gene correction in cells derived from patients with inherited disorders. We sought to explore the potential of CRISPR/Cas9 genome editing to correct acquired mutations in human leukemia cells and to determine the impact of mutation correction on cellular function.

In this study, we have corrected a specific point mutation in *ASXL1*, a gene commonly mutated in myeloid malignancies, in a leukemia cell line using CRISPR/Cas9 technology. CRISPR/Cas9-based correction of the *ASXL1* mutation found in the CML KBM5 cell line restored the expression of the ASXL1 protein which is normally absent in myeloid cell lines carrying homozygous nonsense *ASXL1* mutations [13]. We subsequently performed a series of functional studies showing that the wild-type function of the ASXL1 protein was restored following gene correction. In particular, *ASXL1* mutation correction resulted in restored function of PRC2, including HOXA gene down-regulation and increased global H3K27me3 levels, and in reduced cell growth and increased myeloid differentiation.

The CRISPR/Cas9 system can introduce off-target effects, however no sequence alterations were detected in the top five predicted off-target sites examined in all *ASXL1* mutation-corrected clones identified in our experiments. Whole-genome sequencing would be required for a comprehensive evaluation of off-target effects in mutation-corrected and uncorrected clones. Recent studies using whole-genome sequencing have shown a high specificity of the CRISPR/Cas9 system with low incidence of off-target mutations [31–33].

*In vivo* experiments showed that mice with corrected *ASXL1* have a significantly longer survival than uncorrected control xenografts. *ASXL1* mutations are strongly associated with poorer survival in patients with myeloid malignancies [20]. Our results show that the sole correction of *ASXL1* mutation in leukemia cells (even within a background of *TP53* mutation and BCR-ABL fusion gene) increases survival *in vivo*.

This is the first report describing the use of CRISPR/Cas9 genome editing to perfectly correct acquired driver mutations in human leukemia cells. The therapeutic application of genome editing for gene therapy in primary myeloid cells from leukemia patients might be considered, but would present many challenges. For example, patients often carry multiple mutations in different genes, and therefore correction of several mutations at the same time is required. CRISPR/Cas9 genome editing can be used to correct mutations in several genes simultaneously, as multiple genomic loci can be targeted by co-delivery of a combination of gRNAs to the cells of interest [34–37]. Furthermore, if the mutation correction leads to a growth disadvantage, as in the case of correction of an inactivating mutation in a tumor suppressor gene, then the edited cells would likely be outcompeted by the existing malignant cells. In the context of autologous stem cell transplantation in leukemia, bone marrow ablation prior to infusion of mutation-corrected HSCs may provide a possible option for the application of genome editing technology for the treatment of myeloid disorders in the future.

Human cancers harbor mutations that play a critical role in disease initiation and progression. Our work presents a new strategy to illuminate the impact of oncogenic mutations on cellular function and survival, of particular value for newly identified mutations and mutations for which the impact is poorly understood. Moreover, this study may lay the foundations for a new approach to cancer therapeutics.

## MATERIALS AND METHODS

### Cell lines and culture conditions

KBM5 cells (triploid line) derived from a Ph^+^ CML patient in blast crisis were kindly provided by Dr. Bing Z. Carter (Associate Professor, Department of Leukemia, MD Anderson Cancer Center). KBM5 cells were maintained in Iscove's modified Dulbecco's medium containing 10% heat-inactivated fetal bovine serum, 1% L-glutamine, 100 units/mL penicillin, and 100 μg/mL streptomycin. The human myeloid leukemia K562 cell line and the human megakaryoblastic SET2 cell line were maintained in RPMI 1640 medium supplemented with 10% heat-inactivated fetal bovine serum, 1% L-glutamine, 100 units/mL penicillin, and 100 μg/mL streptomycin.

### DNA extraction

Genomic DNA was extracted using the QuickExtract DNA Extraction Solution 1.0 (Epicentre, QE09050,) following the manufacturer's protocol. Briefly, pelleted cells were resuspended in 20 μL of QuickExtract Solution, incubated at 65°C for 6 min and 98°C for 2 min.

### Validation of *ASXL1* mutation in KBM5 cell line

Genomic DNA extracted from KBM5 cells was amplified and sequenced using forward (5′-AGAGGCAGCAGCAGTGGTGA-3′) and reverse (5′-GAGGCTGCTCCACTAATCTCTCA-3′) primers designed to target the sequence where the *ASXL1* mutation is located (c.2128G > T, p.G710X).

*ASXL1* PCR amplification was performed using the Maxima Hot start Taq DNA polymerase Master Mix (Thermo Scientific) starting from 10 ng of genomic DNA as template, with the following thermal cycling conditions:

**Table:** 

Step	Temperature (°C)	Time	Number of cycles
Initial denaturation	95	4 min	1
Denaturation	95	30 s	35
Annealing	52	30 s	35
Extension	72	30 s	35
Final extension	72	10 min	1

DNA extracted from KBM5 cells was also analyzed using a targeted next-generation sequencing myeloid gene panel, as previously described [23].

### CRISPR vector and single guide RNA (sgRNA) cloning

The pX458 vector (Addgene) expressing Cas9 and containing a cloning site for the sgRNA sequence was digested with *BbsI* (NEB). In order to clone the sgRNAs into the pX458 [pSpCas9(BB)-2A-GFP] vector, we designed two complementary oligos for each sgRNA, adding two overhanging sequences (underlined): 5′-CACCGNNNNNNNNNNNNNNNNNNN-3′ and 3′-CNNNNNNNNNNNNNNNNNNNCAAA-5′. The two complementary oligos were denatured at 95°C for 5 minutes, ramp cooled to 25°C over a period of 45 min to allow annealing, and finally ligated with the linearized pX458. Competent cells were transformed with 2 μl of the ligated plasmid, plated with selection and single colonies were expanded prior to plasmid extraction using Maxiprep kit (Qiagen). The correct insertion of the sgRNA sequences was confirmed using Sanger sequencing.

### Selection of *ASXL1* mutation-corrected KBM5 clones

KBM5 cells were transfected using the Amaxa cell line Nucleofector Kit V (Lonza) according to manufacturer's guidelines. 1 × 10^6^ cells were transfected with 10 μg of pX458 [pSpCas9(BB)-2A-GFP-sgRNA] plasmid and 2.5 μl of 100 μM single-stranded oligodeoxynucleotide (ssODN). GFP positive cells were FACS sorted after 24 hours and plated as single cells in 96-well plates. Cells were incubated at 37°C for two weeks prior to *ASXL1* mutation correction screening: a total of 1,027 colonies derived from single KBM5 cells were sequenced using primers and PCR conditions described above.

The percentages of KBM5 colonies with heterozygous or homozygous *ASXL1* mutation correction obtained for each of the three sgRNAs are as follows:

sgRNA#1: 1.63% homozygous corrections; 2% heterozygous corrections
sgRNA#5: no homozygous corrections; 0.46% heterozygous corrections
sgRNA#25: 1.13% homozygous corrections; 1.4% heterozygous corrections

### Western blot and immunoprecipitation analysis

*ASXL1* mutation-corrected and uncorrected KBM5 cells were lysed using RIPA buffer (Pierce) supplemented with Halt Protease Inhibitor Cocktail (Pierce). Protein concentrations were quantified using BCA Protein Assay KIT (Thermo Scientific), according to the manufacturer's instructions. In each experiment, equal amounts of extracted proteins were separated by SDS-PAGE and blotted using nitrocellulose membrane. Western blots were carried out using the following antibodies: ASXL1 (Novus Biologicals, H00171023-m05), BAP-1 (Santa Cruz, clone 3C11: sc-13576), EZH2 (Active Motif, 39933), Histone H3 lysine 27 trimethyl (Abcam, ab6147), Histone H3 (Abcam, ab1791), SUZ12 (abcam, ab12073) and β-actin (Sigma, A3854) as loading control.

Immunoprecipitation experiments were performed using The Pierce Classic Magnetic IP/Co-IP Kit (Thermo Scientific), according to the manufacturer's instructions. The BAP1 protein fraction was immunoprecipitated using and BAP1 antibody and stained for ASXL1 and BAP1. Anti-ASXL1 and anti-BAP1 antibodies used for immunoprecipitation were the same as the ones used for Western blot experiments as described above.

### Histone extraction

Histones were extracted using Histone Purification MiniKit (Active Motif), according to the manufacturer's instructions. Briefly, cells were pelleted and resuspended in ice-cold Extraction Buffer, transferred to spin columns and histones were eluted using Histone Elution Buffer.

### Quantitative real-time PCR

Total RNA was extracted using TRIzol (Life Technologies) and 1 μg RNA was reverse transcribed using the High Capacity cDNA Reverse Transcription Kit (Applied Biosystems), according to the manufacturer's instructions. The expression levels of *HOXA5, HOXA6*, *HOXA7, HOXA9, HOXA10, HOXA13*, were measured using quantitative real-time PCR. PCR amplifications reactions were prepared using LightCycler 480 Probes Master Mix (Roche). Reactions were performed in triplicate on a Roche LightCycler 96 instrument (Roche Diagnostics). *B2M* was used to normalize for differences in input cDNA. Relative quantification was calculated using the ΔΔCT method. We used the following TaqMan gene expression assays: HOXA5 (Applied Biosystems, Hs00430330_m1), HOXA6 (Applied Biosystems, Hs00430615_m1), HOXA7 (Applied Biosystems, Hs00600844_m1), HOXA10 (Applied Biosystems, Hs00172012_m1), HOXA13 (Applied Biosystems, Hs00426284_m1). For HOXA9 we used a custom-made FAM-TAMRA probe (5′-CCCCATCGATCCCAATAACCCAGC-3′, Applied Biosystems) and primers (Forward: 5′-AAAACAATGCTGAGAATGAGAGCG-3′, Reverse: 5′-TGGTGTTTTGTATAGGGGCACC-3′, Eurofins MWG).

### Cell growth assay

Cells were cultured at an initial density of 1 × 10^4^ / mL, and viable cell counts were determined by trypan blue exclusion for 7 consecutive days.

For the experiments concerning response to imatinib, cell growth of uncorrected and heterozygous (*n* = 3 clones) or homozygous (*n* = 3 clones) *ASXL1* mutation-corrected KBM5 cells treated with various concentrations of imatinib (0.25, 0.5, 1, 1.5 μM) was evaluated at 48 hours.

### Flow cytometry analysis

Cells were washed in phosphate-buffered saline (PBS), and stained with the Fixable Viability Dye eFluor 780 (eBioscience) on ice for 30 min. After washing with PBS, cells were then incubated on ice for 30 min with anti-CD33-Brilliant Violet 570™ (clone WM53, Biolegend) and anti-CD15-Brilliant Violet 650™ (clone W6D3, Biologend). Data acquisition was performed on a BD LSRII instrument (BD Bioscience; Franklin Lakes, NJ, USA) and the data were analysed using FlowJo software version 7.6.4.

### May-Grünwald and Giemsa staining

Cells were cytospun on slides and stained with May-Grünwald and Giemsa solution.

### Off-target sequence analysis

To investigate potential off-target cleavage by Cas9:sgRNA in *ASXL1* mutation-corrected KBM5 clones, we performed targeted Sanger sequencing of the entire coding sequence of the *ASXL1* gene (exons 1–12, primers available on request) as well as the top five off-target sites (obtained from the crispr.mit.edu website) for each sgRNA, using the following primers and conditions:

**Table:** 

	UCSC gene	Locus	Forward	Reverse	Product size	Tm (°C)
**Guide 1**	NM_000189 NM_024344 NM_080429 NM_000322 NM_006223	chr2: +75118439 chr15: −42703613 chr1: −154295508 chr6: +42665654 chrX: +71406302	GCTCATCACTGCTGTGGCCT CACCTGATAATCTCCAGTCTGCTCC TGAGATGGAGTTTCACTCTGTTGCC CTACAGACGTCGCTGGATGGTG GTGGCCCAAGATAATTCTTCCA	GGAATCCACAGGGCAAATGC TTCAGGCATACATGGTGAGCTG CGAGGAACAGGACCAAAGTAGTGTC CCCCTGCCTTGAAATTCTCTTG TGCGCCCAGCTACAATTTTG	626 711 892 816 415	55,56 57,55 58,57 57,56 55,56
**Guide 5**	NM_014939 NM_020198 NM_001005862 NR_002835 NM_001008783	chr18: −29523032 chr17: −61851092 chr17: +37866835 chr8: −122652020 chr6: −137243407	TGAGAACCCGGCCTACAGTT GGGAACAAAAACGCGACATGC TGCCCACTGACTGCTGCCAT CTGAGACCCAACAGGCAGCA AATAGCTGTCGCTAGGATTCCC	TTGTACACACTGGGCCATCG GCTCCTTACCTGTGGCCGAA CGCCACTGCGATAGAGCTAACTAA TCGTCAGTCTGGGCTTACTCGG ATGTTGAGGGAGCCCGAGAA	614 773 828 833 378	53,54 59,56 58,57 56,58 53,55
**Guide 25**	NM_001198774 NM_001136265 NM_173687 NM_007229 NM_001014987	chr1: +151278920 chr1: +19234239 chr8: −144126207 chr22: −43289609 chr16: −28997321	TTGAACTCCTGACCTCATGATCCA CTATGCCCTCCCTTCCTCCTGA TGCAGCTATCTAAGAAGTGCCC AGGTAGGGGAGACAGTATTTGGAGG CTGCCTCACCAGCCCTCTCTTT	CTTGGGATATGCTGTCTTGTTTCC GAGCAGAAAGAGGATGGCCCT TGCTGGTGCCTCTGGACAAA AAACTGGCATAGAGAGGGTCACAA AGAATCCCGCCGGGAAGATG	828 758 410 774 754	57,55 58,56 53,56 57,56 58,59

### Statistical analysis

Statistical analysis was performed using GraphPad Prism Software (GraphPad Software). Differences in gene expression (quantitative real-time PCR) were tested using two-sided paired *t*-test. Differences in cell growth were analyzed using two-way ANOVA. Differences in granulomonocytic differentiation experiments were calculated by Wilcoxon signed-rank test. A *p* < 0.05 was considered significant.

### Mouse xenografts

The animal studies were covered by approval by the animal institute of Albert Einstein College of Medicine under protocol 20150702. Uncorrected KBM5 cells and two homozygous *ASXL1* mutation-corrected KBM5 clones (10 million cells/mouse) were injected via the tail veins into 8–10 week old non-obese diabetic–severe combined immunodeficiency–IL-2Rγ null (NSG) mice (The Jackson Laboratory). Mice were monitored for disease and survival was calculated by Kaplan-Meier analysis from data from two independent experiments. Spleens were collected and weighed and differences in spleen weight were analyzed using *t*-test.
